# Establishing content validity for a composite activities-specific risk of falls scale:linkage between fear of falling and physical activity

**DOI:** 10.1186/s12877-021-02211-z

**Published:** 2021-04-26

**Authors:** Jing X. Wang, Lin Y. Chen, Yan N. Jiang, Ling Ni, Jie M. Sheng, Xia Shen

**Affiliations:** 1 Shanghai YangZhi Rehabilitation Hospital (Shanghai Sunshine Rehabilitation Center), Tongji University School of Medicine, Shanghai, 201619 China; 2 Department of Rehabilitation Sciences, Tongji University School of Medicine, Shanghai, 200092 China; 3 Department of Physical Therapy, Yangzhi Affiliated Rehabilitation Hospital of Tongji University, Shanghai, 201619 China

**Keywords:** Content validity, Physical activity, Fear of falling, Falls, Risk, Disability, ICF, Outcomes assessment

## Abstract

**Background:**

Fear of falling (FoF) and physical activity (PA) are important psychological and behavioral factors associated with falls. No instrument quantifies the link between these two factors to evaluate the risk of falls. We aimed to design a scale linking FoF with PA (Composite Activities-specific Risk of Falls Scale, CARFS) for people with various disability levels.

**Methods:**

First, we designed a questionnaire comprising 40 balance-related activities from the International Classification of Functioning, Disability, and Health (ICF) for a pilot survey. Second, participants were interviewed about their activities-specific FoF degree and PA frequency. The participants comprised 30 community-dwelling older adults, hospitalized patients with strokes, and those with spinal cord injuries, each with different disability levels. Third, the content validity of the items was evaluated twice by 12 experienced rehabilitation professionals: one based on experience and the other on the survey responses. Items with a higher than moderate relevance in both evaluations were included in the CARFS. The panel of professionals discussed and voted on the contribution of FoF and PA on the CARF score. Finally, the scale sensitivity in distinguishing disability levels was analyzed to evaluate the population suitability to the CARFS.

**Results:**

The CARFS included 14 activities. A five-point Likert scale was used to quantify degree of FoF (A) and frequency of PA (B). The CARF score (C), which was determined using the eq. C = A+(4-B) + A × B/2, reflected sensitivity to disability levels in most items.

**Conclusions:**

The CARFS has strong content validity for measuring risk of falls in relation to the FoF and PA of people with various disability levels. It has a potential to provide a guide for designing individualized exercise- and behavior-focused fall prevention programs and enable the precise trtrun 0acking of program effectiveness as a multidimensional outcome measure.

**Supplementary Information:**

The online version contains supplementary material available at 10.1186/s12877-021-02211-z.

## Background

Falls occur commonly in community-dwelling older persons as well as in patients with various disability levels. The impact that falls have can be severe, and may result in disabling injuries, loss of independence, and even death, with this, in turn, leading to increased healthcare utilization [1]. Fall prevention is therefore essential and urgent for the health and wellbeing of people, as well as for the future of health and social care services.

Assessment of risk factors and individualized interventions targeting modifiable risk factors are the key fall prevention strategy [2]. Fear of falling (FoF) and physical activity (PA) are important activity-related psychological and behavioral factors that are associated with falls [3]. FoF has been described as an ongoing concern about falling that ultimately leads to avoiding daily activities [4], and has also been recognized as a loss of confidence in one’s balance abilities [5], which are two different theoretical definitions. The World Health Organization defines PA as ‘any bodily movement produced by skeletal muscles that requires energy expenditure’ [6] – including activities undertaken while working, playing, carrying out household chores, traveling, and engaging in recreational pursuits, − and it underlines its health benefits. FoF alone is harmless unless it leads to sedentary behavior or restriction of important daily activities [3, 7].

FoF commonly occurs after falls [8], but is also experienced by non-fallers who may have been aware of environmental hazards or their own risk factors for falling such as reduced mobility or poor health status [3]. This fear can lead to a highly cautious state or even a PA restriction intended to avoid exposure to the risk of falls and can be considered a prudential response to a real or perceived threat [7]. Taking a bath, going out when it is slippery, and walking several blocks outside are the most noticeably restricted activities in older adults [9]. These activities either have environmental hazards or require a high level of functional balance and mobility, or both. Activities-specific FoF and PA restriction could be beneficial for safety by encouraging caution and reducing risk exposure. However, excessive FoF and long-term PA restriction on various activities result in physical deconditioning, increasing instead the potential risk of falls, which may further fuel FoF and PA restriction [7]. Generally speaking, FoF and PA interact with each other, and both of them play a role in preventing falls, which is initially protective but detrimental in the long term [3, 7].

Therefore, a composite measure evaluation of both activities-specific FoF and PA and the link between them could strengthen the assessment of fall risk and provide a better guide for designing individualised fall prevention programmes, enabling the precise tracking of the programme effectiveness on the psychological and behavioural aspects of fall risk. To the best of the authors’ knowledge, no such instrument is currently available.

The Falls Efficacy Scale (FES) and the Activities-Specific Balance Confidence (ABC) scale are commonly used FoF outcome instruments based on the definition of FoF as loss of balance confidence [4]. The FES assesses one’s degree of perceived self-efficacy at avoiding falls during basic daily activities [10] It shows good psychometric properties for measuring FoF in people with declined functions [10] Meanwhile, the ABC scale assesses one’s degree of confidence in performing daily activities without losing balance or becoming unsteady. The ABC scale items consist of a series of activities with a wide range of difficulties and have good psychometric properties, especially for people with less functional decline [11]. Without the PA information, both scales cannot estimate how FoF affects daily activities.

The Survey of Activities and FoF in the Elderly (SAFE) is another commonly used FoF assessment tool that directly asks the degree of FoF. The SAFE first asks whether the respondent currently does a specific activity. If the answer is yes, it assesses the degree of their FoF and the extent of their restriction compared to 5 y ago; if their answer is no, which indicates total restriction of the specific activity, it assesses the degree of FoF resulting in their total restriction and asks them to specify other reasons, besides FoF, for their total restriction [9]. The SAFE has good psychometric properties for assessing FoF or PA restriction among older adults, but it does not measure the link between FoF and PA restriction [9]. Furthermore, the multiple steps and divided questions make the SAFE suboptimal in quantifying change in follow-up evaluations. Therefore, it is not suitable as an outcome measure.

This study aimed to design a scale linking FoF with PA: the Composite Activities-related Risk of Falls Scale (CARFS). The CARF score was calculated using the degree of FoF, the frequency of PA, and the link between the two. We targeted people with different disability levels, including older adults without any disabilities.

## Methods

### Design

We followed the study design checklist COSMIN (COnsensus-based standards for the selection of health Measurement INstruments) to design this study [12]. The study was divided into four steps: 1) developing an initial questionnaire with potential items and questions, 2) conducting the pilot survey on the targeted population, 3) evaluating content validity from the perspectives of professionals, and 4) assessing content validity through statistical analysis.

We received approval for this study from the ethics committee of Yangzhi Affiliated Rehabilitation Hospital of Tongji University. The study complies with the Declaration of Helsinki.

### Subjects

Older adults without any walking disabilities and persons with stroke or spinal cord injuries (SCI) who had different walking disability levels were targeted as subjects in a pilot survey study, considering that they faced the potential risk of falls due to older age, disease or injury, or both [2, 13, 14]. The walking disability levels were determined with the Functional Assessment Measure (FAM), a scale which evaluates walking independence as follows: no disability (complete independence in a timely, safely manner), slight disability (modified independence with extra time or assistive devices), and severe disability (dependence with supervision or assistance) [15]. Subjects were recruited from the University Affiliated Rehabilitation Hospital and the nearby community using poster advertisements. The selection criteria comprised the following individuals: 1) older adults aged 60 or above and without any walking disability, or with a stroke or SCI diagnosis, 2) who had no communication problem, with a Mini-Mental State Examination score of 24 or above, and 3) who agreed to take a 30-min structured interview. The target sample size for the initial survey was 10 for each subgroup (Table 1).

**Table 1:** Characteristics of target participants who completed the questionnaire survey

Characteristics	Older *N* = 10	Stroke *N* = 10	SCI *N* = 10
Age (year)	64 ± 6	60 ± 13	48 ± 12
Sex (Male: Female)	5:5	8:2	10:0
Walking Independence			
*Dependence*	0	4	8
*Modified Independence*	0	3	2
*Complete Independence*	10	3	0
Paralysis types			
*Hemiplegia*	*0*	*10 (L: R = 4:6)*	*0*
*Paraplegia*	*0*		*5 (Incom:Com = 3:2)*
*Quadriplegia*	*0*		*5 (Incom)*
Months after onset	–	6 ± 2	5 ± 2

*SCI* spinal cord injury, *L* left, *R* right, *Incom* Incomplete, *Com* Complete

Rehabilitation professionals were recruited from the University Affiliated Rehabilitation Hospital by personal contact. The selection criteria included professionals who, 1) were rehabilitation doctors, physiotherapists, occupational therapists, nurses, and other rehabilitation professionals, 2) had a minimum of 5 y of experience in rehabilitation, 3) were currently practising in the field, and 4) were available for two panel meetings of around 1 h each. A total of 12 rehabilitation professionals were invited for content validity evaluation. This group consisted of two doctors, five physical therapists, two occupational therapists, two nurses, and one social worker. Their work experience in rehabilitation ranged from 5 to 28 years, with an average of 10 ± 7 years.

### Procedure

1. *Instrument development*

A structured close-ended questionnaire was designed to ask subjects the following questions: ‘How confident are you regarding not losing balance or falling when you do the following activities?’ and ‘How often did you do the following activities in the past month?’ Their confidence was then scaled using a visual analogy score from 0 to 100 [11]. The frequency used was the real number of times per day for daily activities, times per week for weekly activities, and times per month for non-weekly activities. For daily posture-maintaining activities such as sitting and short-distance walking, which are difficult to report in frequency, PA time was depicted in minutes per day in the survey. The PA time was converted to PA frequency when comparing it with other activities. Considering 10 min as the minimum PA duration of one walking bout in the International PA Questionnaire [16], 10 min were used to estimate the daily PA frequency of these activities in the study, by which PA duration was divided (Table 2). For daily activities, an extra question was asked: ‘Do you think you do these activities more often than normal?’

**Table 2:** Questionnaire responses of subjects

Activity Items	PA frequency (times/month)			*P*^	Balance confidence (0–100)			
	Elderly	Stroke	SCI		Elderly	Stroke	SCI	*P*^
***D4 Mobility***								
Sitting-to-standing	390 ± 232	732 ± 899	207 ± 188	.113	99 ± 3	86 ± 21	50 ± 45	.002
Lying-to-sitting	78 ± 55	303 ± 450	213 ± 142	.202	100 ± 0	90 ± 18	99 ± 3	.061
Bending down-up	285 ± 432	378 ± 925	60 ± 105	.478	97 ± 7	54 ± 40	28 ± 44	.001
Squatting down-up	159 ± 267	36 ± 66	0 ± 0	.082	100 ± 0	67 ± 40	28 ± 44	.000
Kneeling down-up	9 ± 28	6 ± 18	0 ± 0	.590	100 ± 0	67 ± 40	28 ± 44	.000
Sitting activities^@^	1494 ± 730	1926 ± 857	936 ± 500	.016	100 ± 0	100 ± 2	88 ± 20	.045
Standing activities^@^	1620 ± 704	1152 ± 636	141 ± 145	.000	98 ± 4	100 ± 2	48 ± 45	.000
Squatting activities^@^	296 ± 438	42 ± 75	0 ± 0	.033	98 ± 6	70 ± 42	20 ± 38	.000
Transferring while sitting	69 ± 61	204 ± 464	159 ± 162	.569	99 ± 3	80 ± 37	56 ± 49	.039
Lifting and carrying objects	231 ± 184	33 ± 60	6 ± 18	.000	99 ± 3	56 ± 45	23 ± 35	.000
Walking long distances*	18 ± 11	2 ± 4	0 ± 0	.000	97 ± 7	69 ± 30	69 ± 30	.000
Walking short distances^@^	177 ± 227	98 ± 135	10 ± 26	.070	100 ± 0	85 ± 34	32 ± 44	.000
Walking on slope	71 ± 101	13 ± 18	0 ± 0	.029	100 ± 0	83 ± 30	30 ± 44	.000
Walking on wet ground	34 ± 55	0 ± 0	0 ± 0	.033	93 ± 11	79 ± 29	24 ± 32	.000
Walking on uneven ground	15 ± 35	11 ± 15	0 ± 0	.303	93 ± 11	78 ± 29	24 ± 32	.000
Walking on mobile ground	2 ± 3	2 ± 3	0 ± 0	.170	87 ± 14	72 ± 28	21 ± 28	.000
Walking around obstacles	22 ± 33	3 ± 7	0 ± 0	.040	95 ± 11	81 ± 30	28 ± 39	.000
Climbing up and down	120 ± 70	30 ± 37	0 ± 0	.000	100 ± 0	77 ± 31	28 ± 39	.000
Running	0 ± 0	0 ± 0	0 ± 0	–	100 ± 0	59 ± 25	24 ± 33	.000
Jumping	0 ± 0	0 ± 0	0 ± 0	–	76 ± 14	56 ± 23	23 ± 31	.000
Sitting in a wheelchair^@^	0 ± 0	90 ± 142	93 ± 137	.235	100 ± 0	100 ± 0	99 ± 3	.341
Riding bike	6 ± 8	0 ± 0	0 ± 0	.007	78 ± 42	0 ± 0	0 ± 0	.000
Driving motorized vehicles	3 ± 9	0 ± 0	0 ± 0	.381	60 ± 52	0 ± 0	0 ± 0	.000
Using transportation	8 ± 17	1 ± 4	1 ± 1	.221	100 ± 0	78 ± 37	65 ± 46	.085
**D5 Self-Care**								
Washing oneself	12 ± 9	12 ± 11	7 ± 5	.269	99 ± 3	89 ± 28	70 ± 28	.031
Toileting	189 ± 42	144 ± 61	75 ± 104	.007	100 ± 0	89 ± 31	71 ± 20	.019
Putting on-off coats	84 ± 41	72 ± 28	102 ± 63	.371	100 ± 0	90 ± 32	95 ± 11	.519
Putting on-off trousers	72 ± 25	69 ± 20	72 ± 25	.948	100 ± 0	90 ± 32	94 ± 16	.551
Putting on-off footwear	72 ± 25	72 ± 28	123 ± 78	.047	100 ± 0	90 ± 32	90 ± 18	.476
**D6 Domestic life**								
Preparing meals	43 ± 36	0 ± 0	0 ± 0	.000	99 ± 3	56 ± 45	22 ± 36	.000
Tidying up dishes	53 ± 31	7 ± 18	0 ± 0	.000	100 ± 0	53 ± 44	22 ± 36	.000
Cleaning cooking area	54 ± 29	7 ± 18	0 ± 0	.000	99 ± 3	71 ± 39	25 ± 40	.000
Washing clothes	11 ± 12	0 ± 0	0 ± 0	.002	100 ± 0	85 ± 8	72 ± 22	.000
Drying clothes	23 ± 12	0 ± 0	0 ± 0	.000	98 ± 5	100 ± 2	48 ± 45	.000
Cleaning living area	16 ± 12	3 ± 9	0 ± 0	.001	99 ± 3	71 ± 39	25 ± 40	.000
Taking care of animals	12 ± 28	0 ± 0	0 ± 0	.199	98 ± 6	54 ± 40	28 ± 44	.000
Assisting others’ self-care	3 ± 9	0 ± 0	0 ± 0	.381	92 ± 6	46 ± 33	14 ± 30	.000
Assisting others’ movement	0 ± 0	0 ± 0	0 ± 0	–	92 ± 6	26 ± 25	14 ± 30	.000
**D9 Community, Social and Civic life**								
Non-contact sports	0 ± 0	0 ± 0	0 ± 0	–	97 ± 7	65 ± 30	23 ± 35	.000
Contact sports	0 ± 0	0 ± 0	0 ± 0	–	87 ± 12	61 ± 27	18 ± 27	.000

*PA* physical activity, *SCI* spinal cord injury; Both variables data were shown with mean ± standard deviation.

^:the *p* value were the group difference among 3 groups using ANOVA test.

@: The frequency of the posture-maintaining or short-distance walking activities was numbered with the total time divided by 10 min which was used as the minimum PA duration of one walking bout in the International PA Questionnaire [16].

*: For walking long distance, every 1 km was regarded as once according to ICF definition [17]

The questionnaire items all pertained to balance-related activities extracted from ICF categories. The extraction work was completed by three persons (the first two authors and the corresponding author). A total of 40 items were extracted, consisting of 24 mobility activities (ICF code: d4), 5 self-care activities (d5), 9 domestic activities (d6), and 2 activities relating to community, social, and civic life (d9). Some paired activities, such as lying down and sitting up, were combined as one item (e.g. lying down-sitting up) in the questionnaire (Table 2). Prior to the questionnaire, information on participant demographics, disease characteristics, and walking independence levels scaled by FAM was collected [15].

To evaluate content validity, a close-ended form was designed to ask professionals, ‘How relevant do you think the following activities are in correlation with the development objectives of the CARFS?’ Relevance was measured using a five-point Likert scale (0 = not relevant at all, 1 = slightly relevant, 2 = moderately relevant, 3 = very relevant, 4 = extremely relevant; Table 3).

**Table 3:** Results of content validity evaluation of CARFS items

	Content validity (0–4)		If satisfied#
	Experiences-based	Responses-referred	
**D4 Mobility**			
Sitting-to-standing	3.7 ± 0.5	3.8 ± 0.4	Y
Lying-to-sitting	1.8 ± 1.4	3.2 ± 0.9	
Bending down-up	2.3 ± 1.4	3.3 ± 0.6	Y
Squatting down-up	2.8 ± 0.8	1.9 ± 0.9	
Kneeling down-up	2.7 ± 1.0	1.8 ± 0.6	
Sitting activities	2.0 ± 1.5	1.9 ± 1.0	
Standing activities	2.8 ± 1.0	2.4 ± 1.1	Y
Squatting activities	3.0 ± 1.0	2.8 ± 1.1	Y
Transferring while sitting	3.2 ± 1.0	3.3 ± 1.0	Y
Lifting and carrying objects	2.7 ± 1.1	2.0 ± 1.0	
Walking long distances	3.3 ± 1.0	2.3 ± 1.4	Y
Walking short distances	2.7 ± 1.2	2.4 ± 1.2	Y
Walking on slope	2.8 ± 1.0	2.0 ± 1.0	
Walking on wet ground	3.6 ± 1.0	2.1 ± 1.4	Y
Walking on uneven ground	3.7 ± 0.5	2.2 ± 1.4	Y
Walking on mobile ground	3.5 ± 0.7	2.0 ± 1.3	
Walking around obstacles	3.3 ± 1.1	2.0 ± 1.5	
Climbing	2.7 ± 1.5	1.8 ± 1.5	
Running	3.2 ± 1.3	1.6 ± 1.4	
Jumping	3.0 ± 1.3	1.7 ± 1.4	
Sitting in a wheelchair	1.7 ± 1.4	2.3 ± 1.4	
Using transportation	2.8 ± 1.7	2.7 ± 1.4	Y
Riding bike	2.8 ± 1.1	1.7 ± 1.4	
Driving motorized vehicles	0.8 ± 1.0	0.4 ± 0.5	
**D5 Self-Care**			
Washing oneself	2.8 ± 1.1	2.7 ± 1.2	Y
Toileting	3.0 ± 1.2	3.5 ± 0.7	Y
Putting on-off coats	1.4 ± 1.3	2.4 ± 1.1	
Putting on-off trousers	2.4 ± 1.3	2.8 ± 1.1	Y
Putting on-off footwear	2.7 ± 1.0	2.9 ± 0.9	Y
**D6 Domestic life**			
Preparing meals	1.9 ± 1.2	1.3 ± 0.8	
Tidying up dishes	1.4 ± 1.3	1.4 ± 1.0	
Cleaning cooking area	1.9 ± 1.2	1.2 ± 0.9	
Washing clothes	1.7 ± 1.4	0.8 ± 0.8	
Drying clothes	2.5 ± 1.2	1.0 ± 1.3	
Cleaning living area	2.3 ± 1.1	1.4 ± 1.4	
Taking care of animals	1.9 ± 1.2	0.8 ± 1.4	
Assisting others’ self-care	2.2 ± 1.2	1.2 ± 1.1	
Assisting others’ movement	2.4 ± 1.2	0.5 ± 1.0	
**D9 Community, Social and Civic life**			
Non-contact sports	2.6 ± 1.5	1.0 ± 1.3	
Contact sports	3.2 ± 1.3	1.0 ± 1.5	

*CARFS* Composite Activities-specific Risk of Falls Scale

#: satisfied if the mean score of content validity was higher than 2 judged in both evaluations

An open-and close-ended evaluation form regarding the questions and scoring manner of the CARFS was also designed to ask the professionals, ‘How much do you think the content correlates with the development objectives of the CARFS?’ Suitability was scaled using the Likert scale as well. Suggestions were required when a score of less than four was given.

2) *Pilot survey*

The survey questionnaires were administered individually to the subjects through standardized face-to-face interviews. We explained the procedures to each of the participants and obtained informed consent written by them prior to data collection.

3) *Content validity evaluation through panel meetings*

Before the panel meetings, we made a series of preparations. A descriptive statistics table of the survey responses of the subject groups (Older, Stroke, and SCI) was made to use as a reference in the panel meetings (Table 2).

Twelve rehabilitation professionals participated in a face-to-face panel meeting. The development objectives, their duty, and the meeting procedure were explained in advance. They were required to scale the relevance of each item in a content validity evaluation form. Double evaluations were conducted by each professional: one based on experience and the other with reference to the survey responses. In both evaluations, the same instructions were given: ‘Please comprehensively consider your own perception of the risk of the subject falling when they do specific activities as well as note their own FoF (loss of balance confidence) and PA frequency when you evaluate the relevance of each item.’

Items with a higher than moderate relevance in both evaluations were included in the CARFS. The other panel meeting was held online to discuss the other details of the CARFS, including questions and manner of scoring.

4) *Content validity evaluation through statistical analysis*

Once the CARFS was developed, the survey responses to the CARFS items were extracted and re-scored in the CARFS format. The validity of CARFS scoring across different levels of walking disability was further assessed through statistical analysis.

### Analysis

SPSS 21.0 software was used for data analysis. Analysis of variance (ANOVA) was used to compare FoF, PA, and CARF scores among three subject groups and across three levels of walking disability. Pearson’s correlation was used to explore the correlations between FoF, PA, and CARF score. The significance level was set at 5%.

## Results

A total of 30 subjects completed the questionnaire survey, with 10 persons in each subject group. Among the 30 subjects, 13 had no walking disability (completely independent), 5 had a slight walking disability (modified independent), and 12 had a severe walking disability (dependent) (Table 1).

### Responses to the questionnaire

Most activities showed a group difference with respect to frequency of PA and degree of FoF that older adults practiced PA more often with less FoF than patients with stroke and SCI, overall. The activities with a higher frequency of PA, or those with a higher FoF (lower balance confidence) than average within each group were highlighted for the professionals when they evaluated content validity. (Table 2).

### Content validity evaluation through panel meetings

Fourteen items were included in the CARFS (Table 3).

After discussing and modifying the CARFS, the suitability of the questions and the scoring description of FoF and PA were all given a full score. The questions on FoF and PA were: ‘How much FoF do you have when you perform the following activities?’ and ‘How often did you do the following activities in the past month?’ A five-point Likert scale was used to quantify FoF and PA. Balance confidence for each level was provided to further understand FoF. To score the degree of FoF, 0 indicates no FoF at all (100% balance confidence), while 1 to 4 indicates slight FoF (> 80% balance confidence), moderate FoF (> 50% balance confidence), high FoF (> 30% balance confidence), and extreme FoF (≤30% balance confidence). To score PA frequency, 0 indicates none (none in the past month), 1 means occasionally (done in the past month), 2 means sometimes (done weekly), 3 means often (done daily), and 4 means very often (done daily, with even higher frequency than normal).

For the CARF score calculation, considering the potential dual roles of the interaction between FoF (A) and PA restriction (4-B) on preventing falls, a formula for calculating the CARF score (C) was proposed: C = A+(4-B) + A × B/k. A+(4-B) reflects the negative impact of FoF (A) and PA restriction (4-B) on preventing falls and, hence, the propensity of FoF (A) and PA restriction (4-B) to increase the risk of falls. A × B reflects the protective role of PA restriction (lower value of B) in preventing falls via decreasing exposure to the risk of falls and then a decrease in the risk of falls resulting from FoF. 1/k was a coefficient that balanced the weight of these dual roles in preventing falls. The k was set in a range of 1 to 4 matching the range of B. The CARF scores, calculated through formulae using different k values, were shown in Table 4. With reference to Table 4, all professionals chose the CARF score calculated using a formula with k equal to 2 as this was the value that manifested the most reasonable correlations with FoF and PA levels. Therefore, the final calculation formula of the CARF score is C = A+(4-B) + A × B/2. The CARF score for each item ranged from 0 to 12 (Table 4). The total score ranged from 0 to 168.

**Table 4:** The corresponding relations among FoF, PA, and CARF scores calculated using different formulae

Factors	FoF	PA	CARF (1)^	CARF (2)^*	CARF (3)^	CARF (4)^
	0	4	0	0	0	0
Scores	0	3	1	1	1	1
	0	2	2	2	2	2
	0	1	3	3	3	3
	0	0	4	4	4	4
	1	4	5	3	2.3	2
	1	3	5	3.5	3	2.75
	1	2	5	4	3.7	3.5
	1	1	5	4.5	4.3	4.25
	1	0	5	5	5	5
	2	4	10	6	4.7	4
	2	3	9	6	5	4.5
	2	2	8	6	5.3	5
	2	1	7	6	5.7	5.5
	2	0	6	6	6	6
	3	4	15	9	7	6
	3	3	13	8.5	7	6.25
	3	2	11	8	7	6.5
	3	1	9	7.5	7	6.75
	3	0	7	7	7	7
	4	4	20	12	9.3	8
	4	3	17	11	9	8
	4	2	14	10	8.7	8
	4	1	11	9	8.3	8
	4	0	8	8	8	8

*FoF* fear of falling, *PA* physical activity, *CARFS* Composite Activities-specific Risk of Falls Scale.

^: CARF value is calculated by the FOF (A) and PA (B) values using a formula of C = A+(4-B) + A × B/k, 1/k is a weight coefficient, which was 1 for CARF (1), 1/2 for CARF (2), 1/3 for CARF (3), 1/4 for CARF (4).

*: the one with the most reasonable correlations with FoF and PA all professionals chose

The CARF score in gray cells is only determined by degree of FoF but not affected by frequency of PA.

### Content validity evaluation of CARFS scoring

The CARF score showed sensitivity to the disability levels in most items (*P* < 0.05) (Table 5). The overall CARF score showed a strong correlation with the degree of FoF (r = 0.855) and a moderate correlation with the frequency of PA (r = −0.557) in all subjects.

**Table 5:** Sensibility analysis of final CARFS factors

	FoF				PA				CARF			
Items	Independent	Modified Independent	Dependent		Independent	Modified Independent	Dependent		Independent	Modified Independent	Dependent	
	*N* = 13	*N* = 5	*N* = 12	*P*^^^	*N* = 13	*N* = 5	*N* = 12	*P*^^^	*N* = 13	*N* = 5	*N* = 12	*P*^^^
Sitting-to-standing	0.2±0.6	0.4±0.9	2.4±1.6	.000^*^	3.1±0.3	3.2±0.4	2.4±1.5	.187	1.5±1.6	1.8±2.4	6.4±3.5	.000^*^
Bending down-up	0.5±0.8	1.8±1.8	3.5±1.2	.000^*^	3.1±0.3	2.6±1.5	0.8±1.4	.000^*^	2.1±2.0	4.7±4.0	7.5±2.3	.000^*^
Standing activities	0.2±0.4	0.4±0.9	1.9±1.9	.005^*^	3.1±0.3	3.4±0.5	2.5±1.9	.324	1.3±1.0	1.8±2.4	4.5±4.1	.024^*^
Squatting activities	0.3±0.8	1.4±1.7	3.3±1.6	.000^*^	1.7±1.7	0.0±0.0	0.5±1.2	.030^*^	2.8±2.1	5.4±1.7	6.8±2.7	.001^*^
Transferring while sitting	0.2±0.6	1.2±1.8	1.8±2.0	.041^*^	2.2±1.5	2.4±2.2	2.1±1.9	.945	2.2±1.7	3.6±3.6	4.1±3.8	.309
Walking long distances	0.7±0.9	1.6±0.9	3.5±0.9	.000^*^	1.8±1.3	0.4±0.9	0.0±0.0	.000^*^	3.2±2.2	5.2±1.8	7.5±0.9	.000^*^
Walking short distances	0.0±0.0	0.4±0.9	2.8±1.7	.000^*^	3.0±0.0	3.0±0.0	1.0±1.5	.000^*^	1.0±0.0	2.0±2.2	6.2±3.2	.000^*^
Walking on wet ground	0.8±0.9	1.4±0.9	3.1±1.3	.000^*^	1.1±1.5	0.0±0.0	0.0±0.0	.024^*^	3.7±2.2	5.4±0.9	7.1±1.3	.000^*^
Walking on uneven ground	0.7±0.9	1.4±0.9	3.2±1.1	.000^*^	1.1±1.5	1.0±1.4	0.3±0.9	.248	3.7±2.0	4.6±2.2	7.0±1.4	.000^*^
Using transportation	0.3±0.8	1.2±1.8	1.5±1.9	.145	1.4±1.3	0.4±0.9	0.3±0.8	.039^*^	3.1±1.8	4.8±2.3	5.3±2.1	.026^*^
Washing oneself	0.3±0.6	1.6±2.2	1.2±1.3	.108	2.6±1.0	1.6±0.9	1.8±1.0	.074	2.2±1.7	4.8±3.9	4.1±2.8	.089
Toileting	0.0±0.0	0.4±0.9	1.5±1.3	.001^*^	3.2±0.4	3.2±0.4	1.8±1.9	.032^*^	0.8±0.4	1.8±2.4	4.3±3.5	.005^*^
Putting on-off trousers	0.0±0.0	0.0±0.0	0.7±1.4	.143	3.2±0.4	3.0±0.0	3.3±0.5	.474	0.8±0.4	1.0±0.0	2.4±3.6	.222
Putting on-off footwear	0.0±0.0	0.4±0.9	0.8±1.4	.174	3.2±0.4	3.2±0.4	3.4±0.5	.334	0.8±0.4	1.8±2.4	2.7±3.8	.236
Total score	4.2±4.9	13.6±11.6	31.2±13.9	.000^*^	33.5±4.9	27.4±5.9	20.2±8.7	.000^*^	29.3±11.0	48.7±23.5	76.0±24.8	.000^*^

*FoF* fear of falling, *PA* physical activity, *CARFS* Composite Activities-specific Risk of Falls Scale

All variables data were shown with mean ± standard deviation. ^^^all results were analyzed through ANOVA

## Discussion

This is the first study to design an activities-specific fall risk scale that quantifies the link between FoF and PA. The CARFS is also the first to target people with different levels of disability, including older adults and persons with neurological disorders. Adopting the ICF system to determine the activity items and the scientific design procedure ensured excellent content validity of the CARFS.

The CARFS uses two questions to determine a subject’s degree of FoF and PA frequency using a five-point Likert rating scale. It is simple to administer. The CARF score calculation links the degree of FoF and PA frequency, considering the dual effects of both on fall risk. The respective relations of the CARF score with the degree of FoF and PA frequency have been recognized by all professionals of the panel and might have the potential to provide clear guidance in designing exercise-and behaviour-related fall prevention protocol in particular. For activities associated with slight FoF or lower, active PA is a protective behaviour that can prevent falls. For activities associated with moderate FoF, active PA does not produce negative effects on fall prevention; therefore, it may be encouraged, but further precaution or preventive intervention is necessary in such cases. For activities associated with high FoF, an inactive PA is recommended to prevent falling or the adoption of alternative means of performing such tasks, unless the FoF improves enough through restorative intervention. For activities associated with extreme FoF, total avoidance can significantly reduce the risk of falls. This guidance is proposed to be applicable to persons with physical functions including balance abilities, matched to their FoF [18]. For specific cases where there is a mismatch between physical functions and FoF, with a prevalence of around 30% in community-dwelling older persons, it is not suitable to modify PA behaviour simply based on the CARF score [19]. On the contrary, psychological or cognitive intervention may be more suitable for them [19].

The suggestions based on the CARF scores are consistent with real phenomena and the professionals’ conceptions. Taking the example of Parkinson’s disease, falls are most common in relatively early disease stages when patients are sufficiently mobile but with postural instability. Conversely, falls occur less in the later stages, when patients are bedridden [20]. In the Morse Fall Scale, which is commonly used in hospitals, unique activity-related risk is scaled as the lowest risk factor in both normal patients and those on bed rest [21].

The scoring of FoF in the CARFS integrates the strengths of two conceptually different measures of FoF [9–11]. A Likert scale was determined to quantify FoF and PA by the panel professionals, which was thought more suitable than 100 scale or 10 scale with a visual analogy score due to the characteristic of PA frequency. The Likert scale is usually used to quantify the FoF directly [9], while a visual analogy score using 100 scale or 10 scale is commonly adopted to quantify balance confidence or efficacy [10, 11]. The two conceptually different measures of FoF showed to be strongly correlated [9], but the balance confidence measure showed a stronger correlation with balance abilities and activity restriction than the direct FoF measure [18]. Therefore, taking into account both the accustomed scoring manner and the stronger correlation with balance abilities and self-restriction, balance confidence for each level was provided for extra-dimensionally understanding FoF. Each point of the Likert scale for FoF has a corresponding degree of balance confidence. The corresponding relation was generated based on cut-off points found in previous studies. An ABC score of 80% has been used as a cut-off point to discern the differences in fall history, balance and mobility, and quality of life [11, 22]. ABC scores of 50 and 30% have been used to differentiate disability levels [11, 23]. Based on those findings, the CARFS is intended to correlate with existing FoF scales. However, further studies are needed to verify the concurrent validity.

Among the multidimensional factors of the CARFS, the CARF score showed a higher correlation with FoF than with the PA frequency. This concurs with the findings of previous studies that used separate scales targeting these factors. Previous studies demonstrated that, compared with PA, FoF correlated more strongly with physical functioning [24], which is a direct risk factor of falls [7].

The CARFS contains a wide range of activities, which cover mobility and self-care categories. The adaptation of the ICF system in its development ensures the comprehensiveness of the activity items. Further, double evaluations were used to minimize selection bias and ensure content validity. The descriptions of each item, based on the ICF system, were also easy to understand and explain. Most items showed sensitivity to different disability levels and populations with regard to the degree of FoF, frequency of PA, and CARF score. Therefore, we submit that this instrument may be suitable for use in a wide range of populations, including, but not limited to, community-dwelling older adults and patients with neurological disorders at different disability levels but without a communication impairment.

Although the CARFS demonstrates strong content validity, this study still faces limitations due to the methodology of the pilot survey. First, the survey was only conducted in community-dwelling older adults and patients recovering from strokes and SCI. A small sample size in the pilot survey weakened the representativeness of the target population in both demographic and disease-specific characteristics. Second, the formula of the CARF score was proposed based on professional perspectives but not derived from existing formulae. Third, although we arranged dual evaluations to minimize the risk of selection bias, content validity was evaluated subjectively by professionals. Therefore, it is crucial to evaluate the objective psychometric properties of the CARFS before using it, where larger populations are required to ensure the representativeness of the targeted population. Fourth, as assistive information to guide individuals when scaling degree of FoF, cut-off points of balance confidence were determined based on the cut-off points for discriminating fall history [11, 22] and disability levels [11, 23]. Analysing the direct correlation between balance confidence and the degree of FoF of the CARFS items is necessary to affirm or optimise the cut-off points of balance confidence in the future.

## Conclusion

This study has scientifically developed a scale, the CARFS, that measures the risk of falls in relation to the FoF and PA in people with various levels of disability. It has strong content validity, as judged by rehabilitation professionals, and has been approved through sensitivity tests based on the pilot survey responses. The CARFS has the potential to strengthen the screening process for fall risk, provide better guidance for designing individualised exercise- and behaviour-focused fall prevention programmes, and enable the precise tracking of programme effectiveness as a multidimensional outcome measure. However, further reliability and validity studies are needed.

## Supplementary Information

**Additional file 1.**

## Data Availability

The datasets used and/ or analysed during the current study are available from the corresponding author on reasonable request.

## Abbreviations

FoF: Fear of Falling
PA: Physical Activity
CARFS: Composite Activities-specific Risk of Falls Scale
ICF: International Classification of Functioning, Disability, and Health
FES: Falls Efficacy Scale
ABC: Activities-Specific Balance Confidence scale
SAFE: Survey of Activities and FoF in the Elderly
SCI: Spinal Cord Injuries
FAM: Functional Assessment Measure
ANOVA: Analysis of Variance

## References

[1] Florence CS, Bergen G, Atherly A, Burns E, Stevens J, Drake C (2018). Medical costs of fatal and nonfatal falls in older adults. J Am Geriatr Soc.

[2] Avin KG, Hanke TA, Kirk-Sanchez N, McDonough C, Shubert TE, Hardage J, Hartley G, Academy of Geriatric Physical Therapy of the American Physical Therapy Association (2015). Management of falls in community-dwelling older adults: clinical guidance statement from the academy of geriatric physical therapy of the American Physical Therapy Association. Phys Ther.

[3] Moreira NB, Rodacki ALF, Pereira G, Bento PCB (2018). Does functional capacity, fall risk awareness and physical activity level predict falls in older adults in different age groups?. Arch Gerontol Geriatr.

[4] Tinetti ME, Powell L, Fear of falling and low self-efficacy: a case of dependence in elderly persons. J Gerontol. 1993; 48 Spec No: 35–38. 10.1093/geronj/48.special_issue.35 8409238

[5] Maki BE, Holliday PJ, Topper AK (1991). Fear of falling and postural performance in the elderly. J Gerontol.

[6] World Health Organization. Global Recommendations on Physical Activity for Health [online]. Available at: https://www.who.int/health-topics/physical-activity#tab=tab_1. Accessed 2020/8/5. 26180873

[7] Delbaere K, Crombez G, Vanderstraeten G, Willems T, Cambier D (2004). Fear-related avoidance of activities, falls and physical frailty. A prospective community-based cohort study. Age Ageing.

[8] Friedman SM, Munoz B, West SK, Rubin GS, Fried LP (2002). Falls and fear of falling: which comes first? A longitudinal prediction model suggests strategies for primary and secondary prevention. J Am Geriatr Soc.

[9] Lachman ME, Howland J, Tennstedt S, Jette A, Assmann S, Peterson EW (1998). Fear of falling and activity restriction: the survey of activities and fear of falling in the elderly (SAFE). J Gerontol B Psychol Sci Soc Sci.

[10] Tinetti ME, Richman D, Powell L (1990). Falls efficacy as a measure of fear of falling. J Gerontol.

[11] Powell LE, Myers AM (1995). The Activities-specific Balance Confidence (ABC) Scale. J Gerontol A Biol Sci Med Sci.

[12] Mokkink LB, Prinsen CA, Patrick DL, et al. COSMIN Study Design checklist for Patient-reported outcome measurement instruments [online]. Available at: https://www.cosmin.nl/wp-content/uploads/COSMIN-study-designing-checklist_final.pdf.

[13] Goto Y, Otaka Y, Suzuki K, Inoue S, Kondo K, Shimizu E (2019). Incidence and circumstances of falls among community-dwelling ambulatory stroke survivors: a prospective study. Geriatr Gerontol Int.

[14] Matsuda PN, Verrall AM, Finlayson ML, Molton IR, Jensen MP (2015). Falls among adults aging with disability. Arch Phys Med Rehabil.

[15] Donaghy S, Wass PJ (1998). Interrater reliability of the functional assessment measure in a brain injury rehabilitation program. Arch Phys Med Rehabil.

[16] Craig CL, Marshall AL, Sjöström M (2003). International physical activity questionnaire: 12-country reliability and validity. Med Sci Sports Exerc.

[17] World Health Organization. International Classification of Functioning, Disability and Health (ICF). Geneva Switzerland. 2001.

[18] Talley KM, Wyman JF, Gross CR (2008). Psychometric properties of the activities-specific balance confidence scale and the survey of activities and fear of falling in older women. J Am Geriatr Soc.

[19] Delbaere K, Close JC, Brodaty H, Sachdev P, Lord SR, Determinants of disparities between perceived and physiological risk of falling among elderly people: cohort study. BMJ. 2010; 341: c4165, aug18 4, DOI: 10.1136/bmj.c4165. 10.1136/bmj.c4165 PMC2930273 20724399

[20] Bloem BR, Grimbergen YA, Cramer M, Willemsen M, Zwinderman AH (2001). Prospective assessment of falls in Parkinson's disease. J Neurol.

[21] Schwendimann R, De Geest S, Milisen K (2006). Evaluation of the Morse fall scale in hospitalised patients. Age Ageing.

[22] Myers AM, Powell LE, Maki BE, Holliday PJ, Brawley LR, Sherk W (1996). Psychological indicators of balance confidence: relationship to actual and perceived abilities. J Gerontol A Biol Sci Med Sci.

[23] Kressig RW, Wolf SL, Sattin RW, O'Grady M, Greenspan A, Curns A, Kutner M (2001). Associations of demographic, functional, and behavioral characteristics with activity-related fear of falling among older adults transitioning to frailty. J Am Geriatr Soc.

[24] Hornyak V, Brach JS, Wert DM, Hile E, Studenski S, Vanswearingen JM (2013). What is the relation between fear of falling and physical activity in older adults?. Arch Phys Med Rehabil.

