# Supplementary material for: Establishing content validity for a composite activities-specific risk of falls scale:linkage between fear of falling and physical activity
Source: BMC Geriatr. 2021 Apr 26;21:275. doi: 10.1186/s12877-021-02211-z (PMC8077745; doi:10.1186/s12877-021-02211-z)
Supplement: Supplementary file 1 — Additional file 1. [file 12877_2021_2211_MOESM1_ESM.docx]

***Supplementary***

**Composite Activities-specific Risk of Falls Scale (CARFS）**

**Instructions to Participants:**

This scale consists of 14 items of routine physical activities (PA). For each activity item, please imagine doing the specific activity in your usual manner and living context, then indicate the degree of fear of falling (FoF) experienced while doing it and the frequency with which the activity has been done in the past one month, on a scale ranging from 0–4.

When you scale the FoF, please refer to the level of balance confidence in doing the corresponding activity without losing your balance or falling on a scale ranging from 0–100.

A composite CARF score of each activity will be estimated by the degree of FoF and PA frequency of the specific activity you give.

**Composite Activities-specific Risk of Falls Scale (CARFS）**

| Items | **The degree of FoF** | | | | | **The frequency of PA** | | | | |
| --- | --- | --- | --- | --- | --- | --- | --- | --- | --- | --- |
|  | How much FoF do you have when you perform the following activities? | | | | | How often did you do the following activities in the past month?# | | | | |
|  | 0：no FoF at all  (100% balance confidence） | | | | | 0：none  (none in the past month) | | | | |
|  | 1：slight FoF  (>80% balance confidence) | | | | | 1：occasionally  (done in the past month) | | | | |
|  | 2：moderate FoF  (>50% balance confidence) | | | | | 2：sometimes  (done weekly) | | | | |
|  | 3：high FoF  (>30% balance confidence) | | | | | 3：often  (done daily) | | | | |
|  | 4：extreme FoF  (≤30% balance confidence) | | | | | 4：Very often  (done daily, with higher frequency than the normal) | | | | |
|  | 0 | 1 | 2 | 3 | 4 | 0 | 1 | 2 | 3 | 4 |
| 1. Sitting down & standing up |  |  |  |  |  |  |  |  |  |  |
| 2. Bending down & straightening up |  |  |  |  |  |  |  |  |  |  |
| 3. Standing activities (e.g. reaching, preparing meal, hanging clothes) |  |  |  |  |  |  |  |  |  |  |
| 4. Squatting activities (e.g. squatting toileting) |  |  |  |  |  |  |  |  |  |  |
| 5. Transferring while sitting (e.g. moving from a chair to a bed or from a wheelchair to a seat) |  |  |  |  |  |  |  |  |  |  |
| 6. Walking long distances (more than a kilometre) |  |  |  |  |  |  |  |  |  |  |
| 7. Walking short distances (less than a kilometre, around rooms or outside) |  |  |  |  |  |  |  |  |  |  |
| 8. Walking on wet ground (rainy road or wet ground in the kitchen or washing room） |  |  |  |  |  |  |  |  |  |  |
| 9. Walking on uneven ground (e.g. on grass, gravel) |  |  |  |  |  |  |  |  |  |  |
| 10. Using transportation (moving around as a passenger, e.g. taking a car, train, boat, or airplane) |  |  |  |  |  |  |  |  |  |  |
| 11. Washing oneself (washing and drying one's whole body) |  |  |  |  |  |  |  |  |  |  |
| 12. Toileting (the whole process including planning, eliminating human waste, and cleaning oneself afterwards) |  |  |  |  |  |  |  |  |  |  |
| 13. Putting on/taking off trousers |  |  |  |  |  |  |  |  |  |  |
| 14. Putting on/taking off footwear |  |  |  |  |  |  |  |  |  |  |

#：If the time after disease/ injury onset is less than one month. The time after onset is used to estimate the frequency.
